# A Three-Feature Model to Predict Colour Change Blindness

**DOI:** 10.3390/vision3040061

**Published:** 2019-11-10

**Authors:** Steven Le Moan, Marius Pedersen

**Affiliations:** 1 Department of Mechanical and Electrical Engineering, Massey University, 4410 Palmerston North, New Zealand; 2 Department of Computer Science, Norwegian University of Science and Technology, 2815 Gjøvik, Norway; marius.pedersen@ntnu.no

**Keywords:** change blindness, attention, saliency, colour, memory, masking

## Abstract

Change blindness is a striking shortcoming of our visual system which is exploited in the popular ‘Spot the difference’ game, as it makes us unable to notice large visual changes happening right before our eyes. Change blindness illustrates the fact that we see much less than we think we do. In this paper, we introduce a fully automated model to predict colour change blindness in cartoon images based on image complexity, change magnitude and observer experience. Using linear regression with only three parameters, the predictions of the proposed model correlate significantly with measured detection times. We also demonstrate the efficacy of the model to classify stimuli in terms of difficulty.

## 1. Introduction

**Figure 1:**
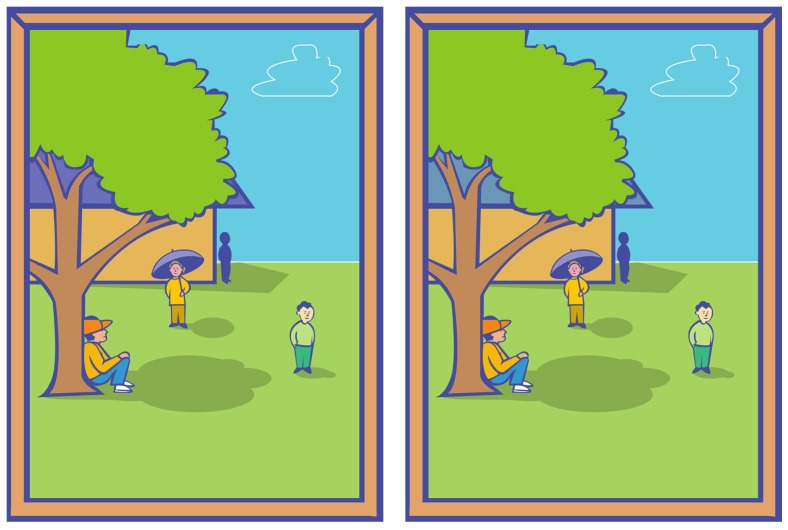
Example of change blindness-inducing image pair.

Despite our impression of a richly detailed visual world, our perceptual experience is surprisingly limited. For instance, consider the ’Spot the difference’ game in Figure 1 (The roof has a different colour.) for the solution). We found that most people need at least 13 s to notice the change under normal viewing conditions. This is an example of change blindness a striking shortcoming of our visual system caused by limitations of attention and memory [1,2,3]. Despite what its name may suggest, change blindness is not a disability, nor is it due to damage to the visual system: everyone is subject to it. It also tends to be significantly underestimated [4], i.e., we see far less than we think we do.

On the one hand, change blindness is known to be responsible for accidents in car traffic [5,6], process plants [7] and even submarines [8], and also for unreliable eyewitness testimony [9]. On the other hand, it has recently been shown that this perceptual failure can be harnessed to reduce computational load in computer graphics [10], to design virtual realities [11], for image compression [12] and visual quality assessment [12]. Change blindness prediction is also useful in visualisation and user interfaces [13,14]. However, the cognitive mechanisms responsible for change blindness are not well understood, which has limited the ability to develop a robust model, particularly for computer graphics and image processing applications.

In this paper, we propose a fully automatic model to predict detection times in image pairs where a single change in colour is introduced during a flicker. The model is based on low-level features that represent the complexity of the scene as well as the magnitude of the change. We also demonstrate that user experience, estimated as the number of stimuli previously viewed, correlates significantly with detection times and we include it in our model. Data were collected via a user study with 66 participants in New Zealand and Norway. We used cartoon images as stimuli as they allow for obtaining more robust scenes and object attributes that are not reliant on noisy low-level feature extractors [15]. A total of 3418 valid detection times were recorded.

After reviewing related work, we describe our experimental design and analyse the results obtained in terms of intra- and inter-observer variability. We then present our model and evaluate its ability to predict detection times as well as to classify “Spot the difference” pairs as either easy or hard.

## 2. Related Work

Early accounts of people’s inability to notice unexpected visual changes in their environment date back to the 19th century [16]. However, it was not before the early 1990s that change blindness research gained real traction, following decades of related work on eye movements and visual memory [1].

Change blindness is a form of high-level visual masking, whereby the change is masked by a disruption and by visual clutter. It is different from low-level masking [17,18] which prevents the perception of a target (e.g., a compression artefact) even though the observer knows where it is, mostly due to limitations of early vision. The disruption that induces change blindness can be abrupt (flicker [1], saccade [19], ‘mudsplashes’ [20], motion change [21]) or gradual [22]. Either way, it is key to preventing exogenous orienting to the change due to bottom-up salience. Instead of using a disruption, one can purposely direct the observer’s attention away from the target to induce another kind of perceptual failure known as *inattentional* blindness. In a famous experiment [23], Simons et al. showed that a person dressed as a gorilla walking among people playing basketball can go completely unnoticed if the observer is focused on counting the number of passes between players. Change blindness and inattentional blindness are both failures of visual awareness. However, they each have a unique background and distinct theoretical implications [2].

The specific causes of change blindness are still debated, but they are known to be linked with attention and memory [3,24]. Visual input data represent about 1 GB/s [25], and it is compressed in the retina to about 8.75 MB/s of raw information [26], which is substantially more than what the brain can handle [27]. Therefore, sensory signals undergo various stages of transformation and selection so they can be processed and interpreted efficiently.

In the visual cortex, spatial pooling over receptive fields of increasing size, latency and complexity [28] is akin to a lossy signal compression, where only the most important information is conveyed from one level to the next. Recent advances in cognitive science [28], brain imaging [29] and machine learning [30] have led to a better understanding of the higher visual cortex, yet there is currently no consensus as to exactly where and how information loss occurs during change blindness [3]. Some researchers support the idea that visual information is only consciously perceived once it is accessed by the attentional functions of late vision, so the cognitive mechanisms associated with awareness, attention, memory and decision-making are intrinsically linked. Others argue that signals emanating from early vision can all reach consciousness but are only partially accessible by the mechanisms associated with decision-making. Both views agree on the existence of a tight bottleneck which researchers from fields such as psychology, cognitive science and philosophy have set out to characterise for over three decades. However, there have been only a few attempts at creating a computational model able to predict high-level visual masking in natural scenes. Existing change blindness models [12,31,32,33,34] based on bottom-up salience and image segmentation are ad hoc and rely on parameter tuning, which is challenging given the paucity of available reference data, particularly for applications related to visual quality.

Change blindness depends on individual factors such as observer experience [35], age [36] and culture [37]. A recent study [38] revealed that detection performance is associated with the ability to form stable perceptual inferences and with being able to resist task-irrelevant distractors. A battery of tests were used to characterise the idiosyncrasy of change blindness in terms of cognitive and attentional capacities. However, for most practical applications of change blindness, such tests are particularly tedious and time-consuming. In this paper, we are interested in predicting change blindness based on stimulus-related factors and limited information about the user.

It has been shown that the masking effect is stronger in visually complex scenes, as they are difficult to encode and maintain in visual short-term memory [3,24,39]. Changes that affect the gist of the scene [40], or which significantly affect bottom-up salience [34] are detected more rapidly. Hou et al. [33] proposed a stimulus-driven predictive model of change blindness based on frequency domain-based salience detection. They compared the sign of discrete cosine transform (DCT) coefficients in the original and changed images and found that the number of unequal signs correlates with detection times. Effectively, this approach allows for estimating the imbalance in salience between the two images. If the changed object is significantly more salient in one image than in the other, the change itself becomes salient and easier to detect. Verma et al. [34] developed a semi-automated approach to generate image pairs with a desired degree of change blindness, based on salience imbalance. Note also that salience imbalance has been shown to predict subjective assessments of image quality in pair comparison setups [41]. Several models have been proposed based on the same precepts, using image segmentation and bottom-up salience to predict change blindness in a semi-automated [32] or fully automated way [31]. However, these methods rely on many intrinsic parameters requiring user input or high-dimensional parameter optimisation. Furthermore, they do not account for the bias due to user experience. Indeed, participants tend to become better at spotting the difference after practising on a few examples and this short-term experience predicts change detection performance.

In this paper, we focus specifically on *colour* change blindness that is the inability to notice difference in colours between stimuli, such as in Figure 1. Colour plays an important role in scene perception [42,43,44], as it is intrinsically linked to the gist of the scene [45]. Nevertheless, large changes in object colours can take more than a minute to be noticed, even if it is in the foreground and even if the observer stares directly at it [46]. The reason why we used exclusively colour-changed image pairs (as opposed to including e.g., objects disappearing or changing position, size, angle) is that having to look for different types of changes is a source of distraction which may reduce performance. We focused on gathering more data for a single type of change.

To that end, we carried out an extensive user study under controlled conditions, which involved 66 participants in two locations: New Zealand and Norway. This paper has two contributions:

- A new publicly available benchmark for change blindness research which includes observer experience.
- A new predictive model of change blindness based on low-level image features and user experience.

## 3. User Study

### 3.1. Participants and Instructions

A total of 66 participants were recruited: 40 in Palmerston North, New Zealand and 26 in Gjøvik, Norway. Ages ranged from 18 to 65 (median: 34.5) and 71% of them were male. All had normal or adjusted-to-normal vision and passed an Ishihara test for colour blindness. They all declared having at least a good command of the English language. Each observer signed a consent form, as per Massey University’s Research Ethics guidelines. They were then instructed as follows (in English):

- Your task is to spot the differences in pairs of images which will be displayed on the screen. Each pair contains a single difference.
- Unlike what you may expect, images will not appear side by side. They will be shown one after the other in a flickering fashion. As soon as you spot the difference, please click on it as quickly as possible. You can click only once. After clicking, the solution will be displayed whether you were correct or not. If after one minute you have not noticed any difference, the solution will be displayed anyway and it will move to the next pair.
- After each sequence of five images, you will have the opportunity to take a short break. Make it as long as you need and feel free to stop the experiment at any time, especially if you feel your focus is drifting away from the task.

The purpose of the latter instruction is to minimise subjective bias due to fatigue, low engagement and negative emotions [47]. Participants viewed 61 stimuli on average (min: 10, max: 100). Image sequences were randomised under two constraints:

- All stimuli had to be seen by an approximately equal number of observers.
- Some scenes had to be seen earlier than others so that the average rank (position in sequence) across all observers follows approximately a uniform distribution. This allowed studying the influence of experience in change detection performance.

This project is approved by Massey University’s Human Ethics Committee (Project identification code: MURF21492, 2 October 2017).

### 3.2. Apparatus

We used Eizo ColorEdge displays (CG2420 in New Zealand and CG246W in Norway), both 61 cm/24.1″ and calibrated with an X-Rite Eye One spectrophotometer (Grand Rapids, MI) for a colour temperature of 6500 K, a gamma of 2.2 and a luminous intensity of 80 cd/m${}^{2}$. Both experiments were carried out in a dark room. The distance to the screen was set to approximately 50 cm (without chin rest). Observers were reminded to keep a constant distance to the screen at the beginning and, when needed, during the experiment.

### 3.3. Stimuli

**Figure 2:**
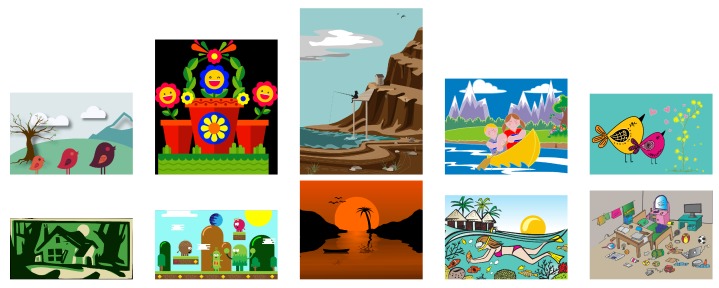
Example scenes from our benchmark.

In total, 100 copyright-free colour images were selected from various online image databases (see Figure 2). We chose exclusively cartoon scenes, as they contain clear, sharp object boundaries, thus making it easier to make seamless changes to specific objects in the scenes. Furthermore, in order to reduce top-down bias due to familiarity with the scenes depicted in the stimuli, we chose them so that they contain no recognisable text, landmarks, characters or cultural symbols of any sort. We also aimed for a balanced a variety of scene complexity (measured with sub-band entropy [48] and edge density [49]). Images also vary in terms of size, with a minimum resolution of 640 × 1000 and a maximum of 1720 × 970. The latter fits on the display with a 100-pixel grey margin on the left and right.

Alternative versions of each of these scenes were created with an image-editing software. The changes made were only in terms of colour (as opposed to moving or removing an object). They affect a single object in each scene, but that object is not necessarily compact and it can be made of several components, e.g., due to occlusion. The minimum magnitude of change was set to 1.2 units of Euclidean distance (median: 5.3, max: 22.3) in the perceptually uniform and hue-linear LAB2000HL colour-space [50], where one unit corresponds approximately to the threshold of just noticeable difference based on the display’s RGB space. The average magnitude over all pairs is about seven units. Changes affect objects of a balanced variety of size, eccentricity and importance (background/foreground). Figure 2 shows examples of stimuli used in the study. The original and modified scenes are respectively noted $s_{i}$ and $s_{i}^{*}$ (i=1,…,100). Each pair $p_{i}=\left\{s_{i},s_{i}^{*}\right\}$ is associated with a decision time for observer *o* noted $T_{o,i}$. Note that there is only one changed version per scene, and it is the same for all observers.

Change blindness was then induced by means of the flicker paradigm [1], with an 800 ms display time and a 80 ms flicker. The stimuli $s_{i}$ and $s_{i}^{*}$ were displayed alternatively for a maximum duration of 60 s or until the change was noticed, whether the first image was $s_{i}$ or $s_{i}^{*}$ was decided randomly at each trial. For each observer, we generated a unique pseudo-random sequence of image pairs. As previously mentioned, the randomness was controlled so as to compensate for the fact that not all observers saw the whole set of 100 pairs. This led to the collection of 34 times per $p_{i}$ on average (min: 21, max: 41) after observer screening.

### 3.4. Screening

Observers were screened based on false positive rate (when a change was reported but far from the changed region) and non-detection rate (when no change was found in less than 60 s). In both cases, any observer with a rate larger than the average plus two standard deviations was considered an outlier. In total, six participants were discarded, three of whom had too high a false positive rate. From visual inspection of the recorded mouse clicks of these participants (recall that observers were asked to click where they saw a change), we found no significant pattern nor any evidence of systematic error.

## 4. Prediction of Change Blindness

### 4.1. Observer Variability and Consistency

**Figure 3:**
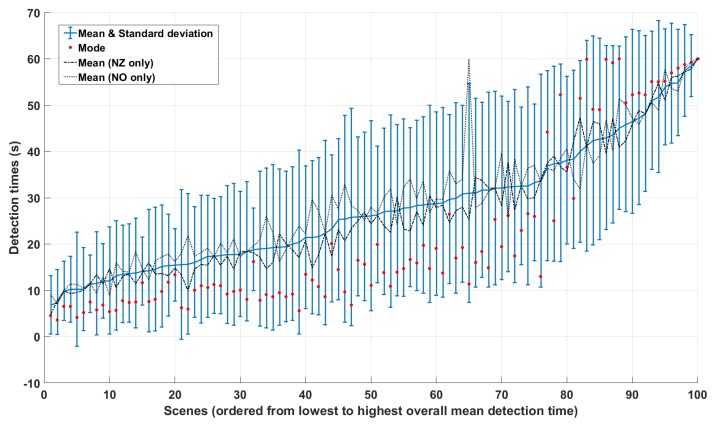
Average (blue line), mode (red dots) and standard deviations (bars) of individual $T_{o,i}$. Notice that, when projecting all the red dots on the *y*-axis, there are two distinct clusters of scenes separated by a region of low density around 35 s. Dotted lines represent the average for the New Zealand and Norway-based observers.

As depicted in Figure 3, observers tend to agree most on which images are the easiest and most challenging ones, since the standard deviations of detection times are the smallest at both ends of the graph.

The fact that change blindness is only temporary makes intra-observer variability difficult to measure. If a person finds a change in a given image pair, any subsequent exposure to the same pair will result in a faster detection. We can, however, analyse how consistently an observer performs with respect to the majority of others. If an observer is systematically slower or faster than all others, this indicates that the variability of this particular observer is smaller than inter-observer variability. For a given *o*, we measured how systematically $T_{o,i}$ was either below or above the mode over all observers noted $\hat{T_{i}}$. A sign test revealed that all of them were consistent in that regard (p<0.01), i.e., each observer is either systematically slower or faster than most. More specifically, 32% of observers were systematically in the fastest 10% while 8% were systematically in the slowest 10% (sign test, p<0.01). This suggests that, on our data, intra-observer variability is small compared to inter-observer variability. We then created two groups of participants: those whose detection times were shorter than $\hat{T_{i}}$ for more than 50% of the stimuli and the rest. This resulted in a group 40 “slow” observers and 20 “fast” ones. It is particularly noteworthy that the average ages are respectively 38.6 and 29.1 years old and that an unequal variances *t*-test (p<0.001) results in the age difference being significant. This suggests a significant effect of age on detection times, which is in agreement with previous studies on individual differences in change blindness [36].

**Figure 4:**
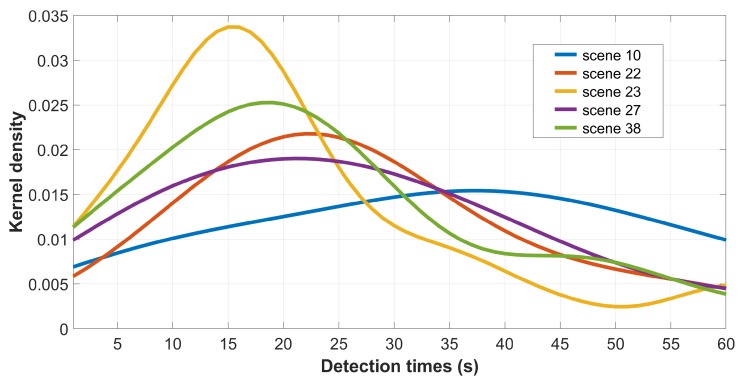
Example of non-normal distributions of detection times. Note: these are kernel density estimates (Gaussian kernel–Silverman rule for bandwidth tuning) from the raw detection times, which are originally in the 1 to 60 s range.

Looking at the distribution of detection times for each scene (recall that we collected at least 15 times per scene), we found that it was normal for 58% of them (Kolmogorov–Smirnov test, p<0.05). Figure 4 depicts representative examples of distributions for the remaining 42%. It can be seen that they are mostly heavy-tailed due to a significant number of observers who either took longer than most to spot the difference or were not able to find it at all.

In view of this, we found that the mode of detection times was more representative than the mean. A univariate kernel density estimate (with bandwidth selection based on Silverman’s rule) was used to estimate the probability density function of each $p_{i}$. All estimates showed one dominant peak, at least twice as high as the next significant peak (if any). The distribution of these dominant modes over the 100 pairs is depicted by the red dots in Figure 3. As opposed to the mean value $\bar{T_{i}}$, $\hat{T_{i}}$ produces two clearly distinct clusters separated by the global density minimum at around 36 s. This led us to the conclusion that the mean detection time is not the most appropriate statistic to represent the degree of change blindness engendered by a given pair (it was used for example in [31]). Rather, we chose the dominant mode $\hat{T_{i}}$ as target value for our regression analysis (see results section). The two naturally emerging clusters C1 and C2 were then used as ground truth for our classification analysis:

$$\begin{array}{cc} \; & C1=\left\{p_{i}|i:\hat{T_{i}}<T_{\mathrm{crit}}\right\},\\ \; & C2=\left\{p_{j}|j:\hat{T_{j}}\geq T_{\mathrm{crit}}\right\}, \end{array}$$

where $T_{\mathrm{crit}}$ is the critical time (minimum density of detection times modes): 36 s.

We also looked at the effect of experience on performance. A significant linear correlation (r=0.41, p<0.001) was measured between $\hat{T_{i}}$ and the average position of $p_{i}$ within the sequence over all observers. This confirms that short-term experience partially predicts decision times.

Note that we did not observe any significant difference between the two groups of participants (NZ and Norway) in terms of decision times, observer variability or prediction performance. Average and mode detection times are both highly correlated between the two groups (Spearman rank order correlations: 0.89 and 0.81, respectively).

### 4.2. Proposed Model

In this paper, our aim is to create a very simple model that is intuitive, computationally efficient and relies on as few parameters as possible. The proposed model is based on three features:

- **Change Magnitude** ($f_{\mathrm{CM}}$),
- **Salience Imbalance** ($f_{\mathrm{SI}}$),
- **User Experience** ($f_{\mathrm{UE}}$).

Note that all stimuli are first converted to the LAB2000HL colour-space [51] before feature extraction.

Change magnitude ($f_{\mathrm{CM}}$) is calculated in terms of pixel colour difference (i.e., the Euclidean distance in LAB2000HL). Only the changed pixels are considered, which effectively means that the size of the change is not accounted for (as opposed to averaging over all pixels within the image):

(1) $$f_{\mathrm{CM}}\left(p_{i}\right)=\frac{1}{\#D}\sum_{D}\Delta E_{00\mathrm{HL}}\left[s_{i}\left(x,y\right),s_{i}^{*}\left(x,y\right)\right],$$

where

(2) $$D=\left\{\left\{x,y\right\}|s_{i}\left(x,y\right)\neq s_{i}^{*}\left(x,y\right)\right\}$$

and $s_{i}\left(x,y\right)$ represents the pixel at spatial coordinates *x* and *y* in $s_{i}$.

**Figure 5:**
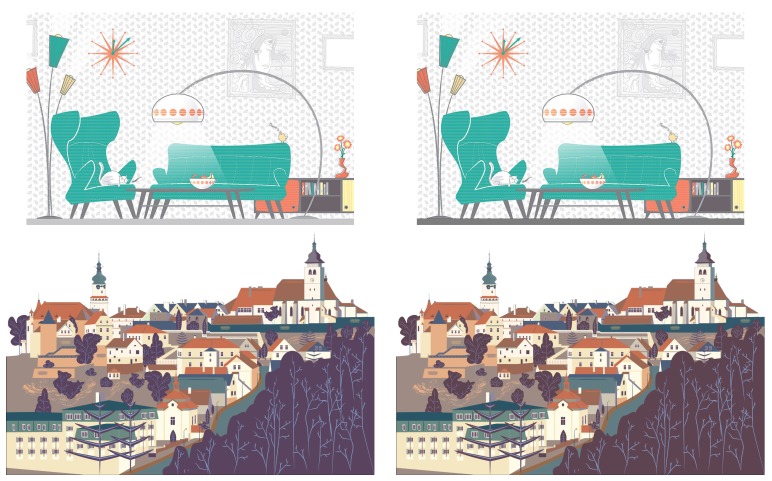
Examples of low salience imbalance but high change magnitude (top) and vice versa (bottom). Note that the top pair has a very large difference, but, at the bottom edge of the scene, which does not immediately attract attention. On the other pair, the difference in color is subtle, but it is over a very large region, which is why it gets picked up by the bottom-up salience features.

Salience imbalance measures how the bottom-up salience maps of the two images differ. The larger the imbalance, the more noticeable the change is. Salience imbalance is different from our change magnitude feature in that the latter does not capture the size or location of the changed pixels. Figure 5 shows examples of low salience imbalance but high change magnitude and vice versa. In this study, it is calculated based on the approach proposed in [33]: the Hamming distance between the signs of Discrete Cosine Transform (DCT) coefficients of $s_{i}$, averaged over its three colour channels:

(3) $$f_{\mathrm{SI}}\left(p_{i}\right)=\frac{1}{3N}\sum_{k}d_{H}\left[\mathrm{sign}\left({\mathrm{DCT}}_{k}\left(s_{i}\right)\right),\mathrm{sign}\left({\mathrm{DCT}}_{k}\left(s_{i}^{*}\right)\right)\right],$$

where $d_{H}\left(A,B\right)$ is the Hamming distance between vectors A and B, *N* is the number of pixels in $s_{i}$ and ${\mathrm{DCT}}_{k}\left(s_{i}\right)$ denotes the discrete cosine transform of the *k*-th channel (either L${}_{00\mathrm{HL}}$, a${}_{00\mathrm{HL}}$ or b${}_{00\mathrm{HL}}$) of $s_{i}$ (same for $s_{i}^{*}$). Note that ${\mathrm{DCT}}_{k}\left(s_{i}\right)$ is in vector form in Equation (3).

Simply put, $f_{\mathrm{SI}}\left(p_{i}\right)$ measures how many 2D frequency sub-bands are significantly affected by the change.

Individual user experience is represented by the index of the image pair within the random sequence generated for each observer (in other words: the number of previously seen examples plus one). The collective experience $f_{\mathrm{UE}}$ associated with the mode of detection times is calculated as the squared average over all observers:

(4) $$f_{\mathrm{UE}}={\left(\frac{1}{N_{o}}\sum_{o=1}^{N_{o}}{\mathrm{id}}_{i,o}\right)}^{2},$$

where ${\mathrm{id}}_{i,o}$ is the index of image pair $p_{i}$ in the sequence generated for observer *o*, and $N_{o}$ is the total number of valid observers (60).

The predicted detection time is eventually obtained via multivariate linear regression:

(5) $$\tilde{T_{i}}=b_{1}f_{\mathrm{CM}}+b_{2}f_{\mathrm{SI}}+b_{3}f_{\mathrm{UE}},$$

where $b_{k}$ (k=1…3) are the only three parameters of the proposed model. In our experiments, these parameters were trained on a portion of the dataset (see the next section). Note that we tried different pooling strategies and found that linear regression gives the best results overall on our data. We also considered a variety of other low-level features such as visual complexity [48,49], alternative salience detection models or image-difference features [52]. We found that they did not help improve the model’s performance.

## 5. Results

We analyse the performance of the proposed model in terms of regression and classification of detection times. Note that we use the following abbreviations: PLCC (Pearson Linear Correlation Coefficient), SROCC (Spearman Rank-Order Correlation Coefficient) and RMSE (Root Mean Square Error).

### 5.1. Regression

We first give, in Table 1, the performance of individual features $f_{\mathrm{CM}}$, $f_{\mathrm{SI}}$ and $f_{\mathrm{UE}}$ as well as age and visual complexity features [48,49]. The latter were calculated in two fashions: globally (accounting for all pixels) and locally (accounting only for pixels within a 20 pixel distance to the changed object/region, including the changed pixels themselves).

We then compare, in Table 2, linear regression to support vector regression, feedforward neural network and decision tree, as well as the model by Ma et al. [31], which we trained and tested on our data. Specifically, we trained the three parameters of Equation (2) in [31] and all other parameters of the model were set as described in the paper. The support vector regression was trained with sequential minimal optimization, and it was given standardised data as input for optimal performance. The neural network was trained with the Levenberg–Marquardt method and with Bayesian regularization. We gave it two neurons, which resulted in an average of 4.8 effective parameters. The decision tree was parametrised to have no more than five splits.

**Figure 6:**
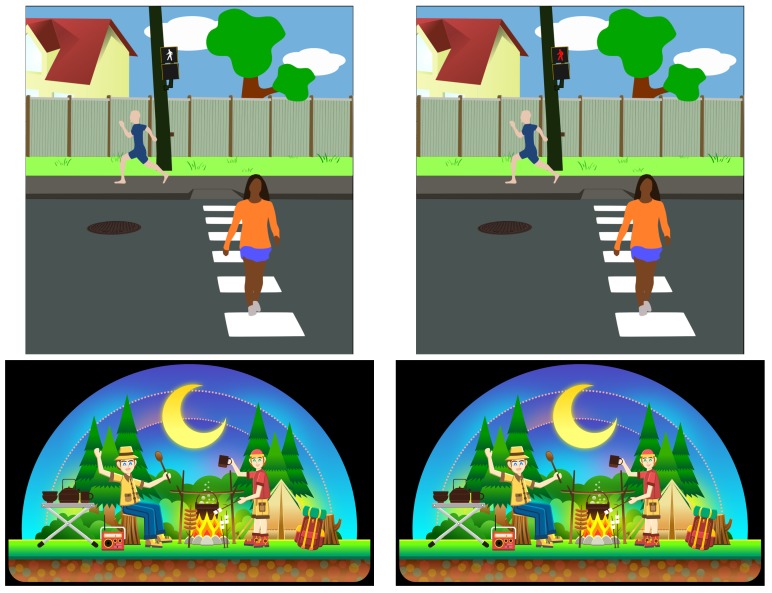
Image pairs corresponding to the lowest (top) and largest (bottom) average detection times. The top scene is arguably subject to a top-down bias that draws attention to the traffic light, resulting in a faster detection. On the other hand, the bottom scene is particularly cluttered and the small magnitude of the change results in virtually no salience imbalance.

Cross-validation was performed by averaging of 100 unique random 70%/30% splits (70% for training and validation, 30% for testing the model parameters). Average Pearson and Spearman correlation coefficients were calculated by first converting the individual *r* to Fisher’s *z* values, averaging them and then converting back to *r*. The significance of the difference between *r* values was performed for each random split individually and averaged (Figure 6).

Furthermore, in Table 3, we report the results obtained when training linear prediction and neural network-based regression models on one group of participants (Norway or New Zealand) and testing on the other. It can be seen that there are no significant differences between the two cohorts.

Overall, the proposed model performs significantly better than any individual feature (including the model by Hou et al. [33]) and than the model by Ma et al. in predicting individual and mode detection times. Interestingly, features that capture visual clutter correlate poorly with detection times. These results also indicate that pooling the proposed features via linear regression gives results that are not significantly worse than via support vector machines, neural network or decision tree with an equal or greater number of parameters. Looking at the optimised parameters of the regression, we found that user experience is the most predictive parameter, followed by salience imbalance and finally change magnitude.

In terms of RMSE, the performance is fairly low all across the benchmark, with a minimum average of 14.8 s to predict mode detection times. This means that a more elaborated model is needed to predict decision times more accurately. Such a model should incorporate individual factors (e.g., cognitive abilities, contrast sensitivity) and high-level attributes (e.g., object importance and semantic categories) to account for top-down biases in visual search strategies. However, in terms of correlation, the proposed model performs well and can rank the 100 image pairs in order of increasing difficulty with reasonable accuracy (SROCC 0.63), especially given its simplicity and the fact that it relies only on three parameters.

**Figure 7:**
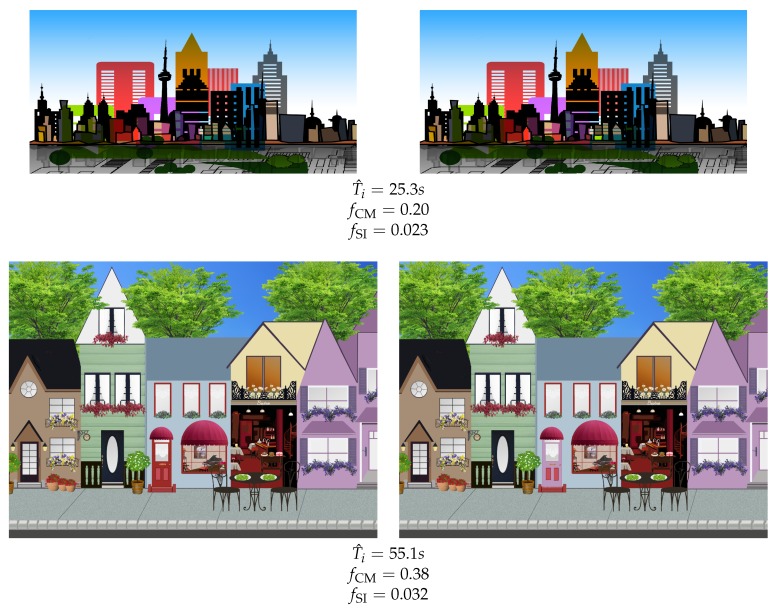
Image pairs corresponding to the best (top) and worst (bottom) prediction of mode detection times (in terms of root mean square error). Solution, top: the green rectangle just at the foot of the red building with vertical stripes, bottom: the central building’s door.

Figure 7 shows image pairs for which the model gave the best and worst prediction of mode detection times (in terms of RMSE). Salience imbalance is nearly identical, but the bottom pair has a larger magnitude of change. However, the latter induces a longer detection time, likely due to more visual clutter. However, neither of the two clutter indices mentioned earlier were able to improve the performance in this case. We believe that a top-down approach to measuring visual clutter is needed to improve performance in such cases.

### 5.2. Classification

Image pairs were classified depending on whether $\hat{T(i)}$ was below or above 35 s (C1 or C2). We used Quadratic Discriminant Analysis to identify these two classes in the three-dimensional space spanned by the model’s features. A ten-fold cross-validation gave an average overall accuracy of 85% to classify mode detection times. Similar performance was obtained with support vector machines and neural network-based classification. Binary tree classification was also implemented, primarily in order to gain insight into the classification process. The resulting tree, where the number of nodes was set to not exceed 4 (for interpretability) yielded an average 0.78 overall accuracy. It shows that the most difficult image pairs are those with either:

- a small $f_{\mathrm{UE}}$ and a large $f_{\mathrm{CM}}$,
- a large $f_{\mathrm{UE}}$, large $f_{\mathrm{CM}}$ and small $f_{\mathrm{SI}}$.

Based on the decision tree, user experience ($f_{\mathrm{UE}}$) is the most predictive feature, as previously noted.

## 6. Conclusions

Change blindness is an intriguing phenomenon that reveals limits in our ability to draw attention and commit information to memory. It is exploited for instance in the famous game ‘Spot the difference’, where participants need to identify one or several differences between seemingly identical scenes and typically take several minutes to do so. Change blindness is influenced by a variety of individual and stimulus-related factors. Understanding its origins and implications can benefit applications such as image coding and quality assessment, where advanced models of visual perception are used to determine what information is more or less important in the scene. While most recent research has focused primarily on modelling early vision in a bottom-up approach, change blindness, as a type of high-level visual masking, cannot be accurately captured by such models. In an effort to develop a computational model of change blindness in natural scenes, we measured the detection times of 60 participants in 100 ‘spot the difference’ games and proposed a simple model to fit our experimental data. Each image pair contained a single change that affected the colour of an object and participants had 60 s to find the change. Our first finding was that the distribution of detection times for a given scene was heavy-tailed in a significant number of cases, indicating that, in these cases, a small number of participants required more time than most to notice the change. This led us to use the dominant mode of detection times rather than the mean, as commonly done in the literature. In line with the results of numerous previous studies, we also found a significant influence of age, experience, as well as the magnitude of the change and how it affects scene content. We then introduced a very simple model based on user experience, change magnitude (colour difference) and salience imbalance. To the best of our knowledge, this is the first model that accounts for short-term experience that is the experience gained throughout the experimental study. Furthermore, unlike existing models such as [31] which is based on seven parameters, ours relies on three parameters only and can predict decision times with significantly better accuracy than individual features and than existing models. The model can be used to rank image pairs in terms of difficulty and to classify as either easy or difficult with an overall accuracy of 85%.

A more advanced model of change blindness will need to predict visual search strategies and eye movements, as well as the probability of encoding. However, this would require more parameter tuning and substantially more data for calibration/training. Arguably, there would also be limits to the accuracy to be obtained (in terms of either RMSE or correlation), due to the stochastic nature of eye movements and visual search strategies. Recent research on saccade and fixation prediction [53] have shown that predicting individual patterns is very difficult, suggesting that individual factors of attention and memory capacity are not yet well understood.

## Figures and Tables

**Table 1:** Prediction performance of individual features. Correlation coefficients in bold font are not significantly different from each other column-wise (*p* < 0.01).

	Indiv ($T_{i}$)		Mode ($\hat{T_{i}}$)	
	PLCC	SROCC	PLCC	SROCC
Change magnitude	0.28	0.30	**0.37**	**0.45**
Salience imbalance (based on [33])	−0.29	−0.29	**−0.39**	**−0.46**
User experience	0.08	0.18	**0.42**	**0.30**
Age	0.19	0.18	/	/
Subband entropy (global)	0.12	0.13	0.15	0.19
Subband entropy (local)	0.16	0.20	0.21	0.21
Edge density (global)	0.08	0.08	0.13	0.12
Edge density (local)	0.10	0.12	0.14	0.15

**Table 2:** Prediction performance of model (average of 100 random 70/30 splits). Correlation coefficients in bold font are not significantly different from each other column-wise (p<0.01).

	Indiv ($T_{i}$)			Mode ($\hat{T_{i}}$)		
	PLCC	SROCC	RMSE	PLCC	SROCC	RMSE
Linear regression	**0.29**	**0.29**	20.0	**0.62**	**0.63**	14.8
SVR	**0.34**	**0.36**	20.2	**0.61**	**0.62**	16.3
NN	**0.36**	**0.35**	19.3	**0.55**	**0.57**	**15.6**
Tree	**0.40**	**0.37**	19.0	0.45	0.41	17.7
Ma et al. [31]	0.11	0.09	35.2	0.39	0.39	22.5

**Table 3:** Prediction performance of model for $\hat{T_{i}}$ (training on one group of participants, testing on the other).

	Training on NZ Data			Training on NO Data		
	PLCC	SROCC	RMSE	PLCC	SROCC	RMSE
Linear regression	0.59	0.56	17.5	0.55	0.55	17.1
NN	0.61	0.60	15.8	0.59	0.58	16.2
Ma et al. [31]	0.04	0.13	39.2	0.29	0.34	24.5
